# Rhythmic displays of female gibbons offer insight into the origin of dance

**DOI:** 10.1038/srep34606

**Published:** 2016-09-30

**Authors:** Peng-Fei Fan, Chang-Yong Ma, Paul A. Garber, Wen Zhang, Han-Lan Fei, Wen Xiao

**Affiliations:** 1 School of Life Sciences, Sun Yat-sen University, Guangzhou 510275, P.R. China; 2 Institute of Eastern-Himalaya Biodiversity Research, Dali University, Yunnan 671000, P. R. China; 3 Department of Anthropology Program in Ecology and Evolutionary Biology, University of Illinois, Urbana, Illinois 61801, United States of America

## Abstract

Dance is a universal art form practiced by all human societies and has many functions including sexual attraction, social cohesion, and the therapeutic release of energy. Dance also has been reported in a small number of non-human primate species, in particular apes. However, its function has not been systematically evaluated. We observed 357 intentional, rhythmic, and nonverbal dance displays performed by four adult female cao vit gibbons (*Nomascus nasutus*) residing in four polygynous groups during 3000 h of observation in Bangliang, Guangxi, China. Females used dance to solicit copulations, as well as to promote a social bond with the group’s lone adult male. In addition, this display appears to represent a form of non-aggressive competition among adult females living in the same group. We found that a female who had a weaker social relationship with the breeding male increased her social and sexual access to the male by an increase in dancing frequency. Given that gibbons dance in various behavioral contexts, and appears to serve several important social and sexual functions, a greater understanding of this form of gestural communication offers an instructive model for examining the origin and evolution of dance in humans and other apes.

Dance is a universal art form practiced by all human societies that employs rhythmic, purposeful, and culturally patterned movements of the body, limbs, and head to communicate nonverbally^1^. Although many functions including sexual attraction, social cohesion, and the therapeutic release of energy have been attributed to dance across human societies^1^,^2^,^3^, the evolutionary origins of dance remain largely unknown. Evolutionary anthropologists have argued that dance in humans is likely to have its origins in the rhythmic movements/displays of non-human primates^3^,^4^.

Dance-like courtship displays have been reported in many species of birds^5^ (detailed descriptions can be found for *Chiroxiphia linearis*^6^ and for *Menura novaehollandiae*^7^). These displays are highly stereotypic^5^ and predictable^7^, and occur almost exclusively during breeding periods^8^. Among non-human primates, rhythmic dance or dance-like behavior so far, has been reported only in apes^9^,^10^,^11^,^12^,^13^,^14^ and white-faced capuchin monkeys (*Cebus capucinus*)^15^. The “rain dance” performed by chimpanzees (*Pan troglodytes*) consists of excited hooting and rhythmic movements in which body mass is transferred from foot to foot, tree branches are swayed, and the displayer runs up and back repeatedly^9^. The “chest beating” display of mountain gorillas (*Gorilla beringei*) involves hooting, rhythmic chest beating and hitting of the ground with both hands^10^. Female Hainan gibbons (*Nomascus hainanus*) performed “a strange rhythmic movement” in which a female extended “her arms out and moved them in a jerky, undulating fashion…. and exhibited rhythmic head-bobbing” that was used to sexually solicit an adult male^12^. A possible instance of dance was performed by siamangs (*Symphalangus syndactylus*)^13^,^14^. Adult and juvenile female siamangs performed “upward thrusts”^13^ or “jerking body movements”^14^ by hanging in front of a male and moved their body up and down by angling their arms repeatedly in sexual and play contexts. In the case of white-faced capuchin monkey (*Cebus capucinus*), females perform a courtship dance that includes a duck-face pirouette and slow motion chasing. During the dance, the male and female maintain this duck face as well as eye contact, and produce either grunts or squeaks^15^. Although the presence of dance-like displays in birds and primates offers a framework for addressing broad evolutionary questions regarding communication and reproduction, given phylogenetic differences in behavioral plasticity, similarities in avian and primate gestural communication are likely to involve different behavioral-neurological pathways and represent parallel solutions to similar socioecological challenges.

Hanna^1^ identified four specific components of dance across human cultures and Francis^3^ concluded, after careful analysis, that patterned movements and displays by chimpanzee’s and gorilla’s met three of the four criteria. Specifically, these displays were purposeful, intentionally rhythmic, and in most instances, nonverbal. The fourth criterion, that dance is culturally patterned could not be evaluated in her review^3^. Francis^3^ also suggested that dance in humans and dance-like behaviors in non-human primates are forms of communication and expression that represent a continuum, with “dance-like” (defined as meeting at least two of the criteria) at one end and “dance” (meeting all four criteria) at the other end. “The continuum demonstrates a gradual progression toward the increased behavioral, cognitive, and cultural complexity that characterizes the dance behavior of human primates”^3^. To avoid confusion, we use the term dance to describe primate rhythmic displays throughout the rest of this paper.

Although functions of human dance such as social bonding, group cohesion, and the initiation of sexual behavior, have been attributed to the dance of non-human primates^3^, rigorous tests of the functions of dance in non-human primates have never been conducted, due in large part to its highly contextual and therefore infrequent occurrence. Here, we present data on 357 rhythmic dance displays performed by four wild female cao vit gibbons (*N. nasutus*) residing in four polygynous breeding groups in the Bangliang Nature Reserve, Guangxi, China. Similar to its close relatives, the western black-crested gibbon (*N. concolor*) and the Hainan gibbon (*N. hainanus*), but unlike other gibbon taxa which are usually characterized by a strong male-female pair bond and social monogamy (e.g. *Hylobates*, *Symphalangus*, *Hoolock*), cao vit gibbons exhibit a polygynous mating system in which groups contain a single adult male and two concurrently breeding females^16^,^17^.

For the first time, we describe, in detail, the individual elements of the dance performed by female cao vit gibbons. To better understand the social and sexual function of this dance, we tested the following hypotheses:

1. Sexual solicitation Hypothesis: if the cao vit gibbon dance display functions principally as an elaborated form of female sexual solicitation for reproduction, than we expect a limited proportion of copulations to occur following a dance display.
2. Social bonding Hypothesis: if dance is used by females in polygynous groups to reinforce a social bond with the lone breeding male, then we expect (2A) a strong positive correlation between the frequency of female dance and the proportion of time that female spends in close proximity (≤1m apart) with the resident male and (2B) after the first dance display on a given day, the dancing female spends a higher proportion of time in proximity to the group’s resident male than she did prior to the dance on that same day.
3. Female Competition Hypothesis. If females use dance as a form of intrasexual competition, then we expect (3A) a non-preferred female to dance more frequently and/or engage in more multi-dancing bouts in order to increase her sexual access to the breeding male compared to a preferred female, and (3B) that females who dance more frequently maintain a closer spatial relationship to the male compared to females who dance less frequently.

## Results

### Description of a single cao vit gibbon dance bout

**Figure 1:**
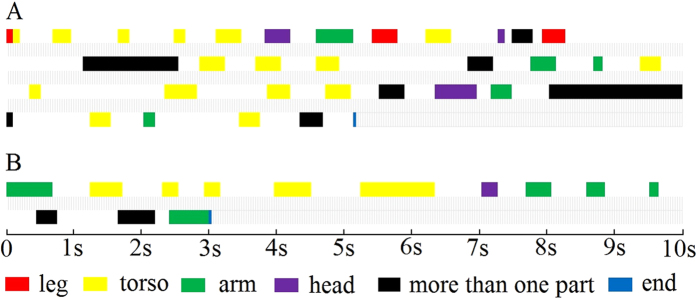
Two dancing displays performed by adult female F11 in G1A, showing a distinct pause between movements of the arms, head, or torso. The first display ended in a copulation whereas the second dance ended in the male and female grooming.

A rhythmic dance display given by an adult female cao vit gibbon in G1 was first observed on June 6, 2008. In total, we observed 357 dancing displays performed by four of six females in our four study groups. The cao vit gibbon dance display involves a series of movements in which an adult female: 1) begins by squatting or standing 0–3 m in front of the adult male, 2) then engages in a rhythmic moving of her body (arms, legs, trunk, and head) slightly while maintaining other body parts motionless (Fig. 1), 3) only minimally shifting her position during the entire dance. She then engages in 4) an obvious pause (dance A: mean 0.68 ± SD 0.57 s, range: 0.1–2.86 s, N = 32; Dance B: mean 0.60 ± SD 0.19 s, range: 0.24–0.90 s, N = 12) that separates individual movements of the arms, legs, head or trunk before continuing (Fig. 1), similar to a human “Robot Dance”. Neither the female nor the male vocalize during the entire display. In 156 instances, we were able to measure the duration of the display. A single dance lasted on average 32 ± SD 27 seconds (range: 3–143s). A short video of dancing by F11 in G1 was submitted as supporting information.

### Dance context and adult male’s reaction

We defined two or more complete dance displays observed within 5 minutes as a multi-dancing bout. If only one display occurred, we termed this a single-dancing bout. The 357 dancing displays were distributed across 242 dancing bouts, including 173 single-dancing bouts and 69 multi-dancing bouts. During the multi-dance bouts, females danced an average of 2.67 ± SD 1.12 times (range 2–7, N = 69).

More than half of the dancing bouts (N = 123 bouts) occurred while the male was resting. Another 83 dancing bouts occurred in response to either the male (N = 24) or the female (N = 59) approaching the other. In the remaining cases, females danced when the male was feeding (7.0%, N = 17) or singing (7.9%, N = 19, Table 1).

In 50 of the dancing bouts (20.7%), the adult male left the vicinity of the dancing female after the dance. In 80 bouts (33.1%), the adult male exhibited no obvious reaction to the dancing female. In the remaining 112 bouts (46.2%), the adult male showed a positive or receptive response (Table 1). Immediately after these dancing bouts the adult male approached the dancing female (2.9%, N = 7), engaged in grooming (21.9%, N = 53), copulated (15.7%, N = 38), or copulated and engaged in grooming in (5.8%, N = 14). Males were more likely to respond positively to a dancing female (e.g. copulate, approach, groom) in approaching and resting situations, but rarely showed positive responses during feeding and singing (*χ*^2^ = 49.316, df = 20, P = 0.004).

### Behavioral differences between females in G1A

The majority of dancing bouts observed (91.7%, N = 242 bouts) occurred in G1A after the replacement of the initial resident male because both adult females in G1A increased their dancing frequency in the presence of the new breeding male (Female F11: from a rate of 0.006 to 0.193 bouts/h; Female F21: from 0.003 to 0.089 bouts/h). Therefore, all subsequent analyses were conducted only on dancing bouts performed in G1A. Sample sizes for groups G1, G2 and G4 were too small to analyze quantitatively (less than 11).

G1A resident females F11 and F21 maintained similar proximity scores with the new male across the entire 17 month study period (F11: 22.5% ± SD 9.8%, range: 4.9–41.1%, N = 17 months; F12: 22.8% ± SD 11.7%, range: 9.6–53.7%, N = 17 months; Mann-Whitney U test: Z = −0.686, P = 0.492). However, the Hinde index revealed that the resident male was more responsible for maintaining close proximity to F21 (Hinde index: −0.190, N = 132) while F11 was more responsible for maintaining close proximity to the male (Hinde index: 0.264, N = 163). Based on Hinde’s criteria, we recognized F21 as the preferred female in this two female breeding group.

We found that the non-preferred female (F11) engaged in a greater number of single dancing bouts (F11 = 110 vs F21 = 49) and a greater number of multiple dancing bouts (F11 = 42 vs F21 = 21) compared to the preferred female (F21). The male responded by copulating with F11 in 31 of 152 dancing bouts (20.4%) and F21 in 17 of 70 dancing bouts (24.3%). The male copulated with F11 21 times when F11 did not dance and copulated with F21 38 times when she did not dance. F11 presented to the male once in the absence of a dance bout but otherwise we observed no obvious signs of solicitation by either males or females during non-dance copulations. Overall, we found that the male was more likely to mate with the preferred female (F21: 38/55) than with the non-preferred female (F11: 21/52) in non-dancing situations (*χ*^2^ = 20.081, df = 1, P < 0.001).

Female cao vit gibbons were observed to dance and copulate during pregnancy, but were never observed to dance or copulate during the first several weeks postpartum (F11: 1 month; F21: 2 months). In the case of both resident females, dancing frequency and copulation frequency did not differ between cycling and pregnancy (F11: dance, Z = −0.159, P = 0.873; copulation, Z = −0.166, P = 0.868, N_cycling_ = 9 months, N_pregnancy_ = 7 months; F21: dance, Z = −0.698, P = 0.485; copulation, Z = −0.465, P = 0.642, N_cycling_ = 8 months, N_pregnancy_ = 7 months).

Excluding the month after parturition, the monthly dancing frequency of the preferred female, F21, was positively correlated with the proportion of time she spent in proximity to the new breeding male (Spearman’s correlation: r = 0.719, P = 0.003, N = 15 months). For female F11, dancing frequency and spatial proximity to the resident male were positively correlated and approached statistical significance (Spearman’s correlation: r = 0.473, P = 0.064, N = 16 months). Overall, there was no correlation between dancing frequency in a given month and male-female proximity in the following month for either adult female (Spearman correlation: F11, r = 0.358, P = 0.190, N = 15; F21, r = −0.125, P = 0.658, N = 15 months). F11 spent significantly more time in proximity to the resident male after her first dance of the day (Wilcoxon Signed Rank test: Z = −1.961, P = 0.005, N = 12 days with > 8 h observations; 15.7% before vs 26.7% after) and more responsible for maintaining proximity to the male after a dance (H index: 0.333, N = 9 before vs 0.545, N = 22, after) compared to prior to dancing. However, this relationship was not significant for F21 (Wilcoxon Signed Rank test: Z = −1.153, P = 0.249, N = 6 days with >8 h observations; 35.7% before vs 55.2% after) and the adult male was more responsible for maintaining proximity to F21 after she danced (H index: −0.222, N = 9 before vs −0.250, N = 8 after) than prior to the dance. We note, however, that the sample size for F21 was very small.

In five cases, the non-preferred female F11 was observed to dance at a distance of 2–3 m from the group’s male when he was in close proximity (≤1 m) to the preferred female (F21). In four of these cases, the adult male showed positive responses to her (approach: 1 case; groom: 1 case; copulate: 1 case; groom and copulate: 1 case). In one case, F21 danced 2–3 m from the male when he was in proximity to F11, and the male approached and copulated with F21. During 11 additional dancing bouts, within two minutes of the first female dancing, the second female responded also by dancing (co-dancing bouts). In these 11 co-dancing bouts, F11 danced first in five cases, F21 danced first in two cases, and we could not distinguish which female danced first in the remaining four cases because these dances appeared to occur simultaneously. There was a trend for females to perform multi-dancing displays during co-dancing bouts (10/22 vs 59/220, *χ*^2^ = 3.903, df = 1, P = 0.048). In 10 of these 11 co-dancing bouts, the adult male did not approach or copulate with either of the adult females. Co-dancing was never observed in the other two stable gibbon groups (G2 and G4) (N = 9 dancing bouts) or in G1 (N = 11 dancing bouts) prior to male replacement (we observed this group prior to male replacement for a period of 18 months). However, sample sizes for these groups were very limited.

Finally, we found a positive correlation when we examined differences in female dancing frequency (F11 dancing frequency minus F21 dancing frequency) and proximity scores (the percentage of time spent in close proximity between F11 and the male minus the percentage of time spent in close proximity of F21 and the male) within a given day (Spearman correlation: r = 0.476, P = 0.010, N = 28 days >8 hours observation) and across months (Spearman correlation: r = 0.671, P = 0.006, N = 15 months before F21 gave birth). These results indicate that dance strongly influenced proximity patterns among adult group members. The more frequently a female danced, the more she maintained close proximity to the breeding male and the less time the remaining female was in close proximity to the breeding male.

## Discussion

We offer the first in depth description of the rhythmic dance display performed by female cao vit gibbons and examine its function in promoting social and sexual bonds in groups containing multiple breeding females. We observed four different females in four social groups to engage in this behavior. The dance performed by female cao vit gibbons is distinct from all other movement patterns that characterize gibbon social interactions. Similar to dance displays performed by chimpanzees^9^,^11^ and gorillas^10^,^11^, female gibbon dance meets at least three of the four criteria that define dance: purposeful, rhythmic, and nonverbal^3^. Unlike the dance displays described in chimpanzees and gorillas, however, in gibbons these are performed exclusively by adult females, directed only towards an adult male, and are associated with sexual solicitation and social bonding. Given difficulties at our site in obtaining fully unobstructed views of the gibbon’s dance, we were not able to quantify individual or group specific variation in the choreographic movements of these dances. However, as indicated in the two videoed dancing displays, whereas the majority of elements of the dance including its rhythm, limited shift in body position (center of gravity), obvious pause between movements, and the absence of associated vocalizations were consistent, other elements such as the sequence of moving body parts were more variable. Variation within gibbon dances may relate to differences in the meaning of the message communicated, the social context of the message communicated (e.g. given by preferred or non-preferred female, female is ovulating or female is pregnant) or possibly differences in ‘tradition’ or ‘dance form’ that is learned by individual females as members of their group or population. We plan to investigate these possibilities in future investigations.

Female cao vit gibbons dance in several behavioral contexts and therefore this form of gestural communication may serve alternative functions. We tested three main hypotheses to explain the function of dancing behavior in cao vit gibbons. We found that the breeding male copulated with a dancing female in 21.5% of cases (20.4% of dancing bouts with F11 and 24.3% of dancing bouts with F21). These results offer support for Hypothesis 1, namely that although not all dances ended in copulation, dancing was used by females, successfully and unsuccessfully, to attract the attention and solicit copulations from the group’s lone breeding males. Like many anthropoid species^18^,^19^, female cao vit gibbons engage in sexual behavior outside of their cycling period. This common primate pattern highlights the function of sexual behavior in both maximizing the likelihood of conception and in establishing and reinforcing an intersexual social bond. Dance appears to represent a form of sexual solicitation that similarly functions to reinforce male-female social bonds.

We next tested the social bonding hypothesis to explain the function of dancing behavior. We found breeding males displayed several affiliative responses to a female’s dance, such as approaching and engaging in grooming. Moreover, after replacement of the breeding male, both females in G1A danced at a considerably higher rate (25 times higher) than either had with their previous breeding partner. We also found that the dancing frequency of both females was positively correlated with their close spatial proximity to the resident male, although the result was not significant for the non-preferred female (P = 0.064). Given that F11 spent significantly more time in close proximity to the male after her first dance of the day compared to prior to her dancing that day, it is likely that dance functioned to strengthen or intensify the sociosexual bond in the short-term. The preferred female F21, also spent a higher proportion of time in proximity to the male (35.7% before dance and 55.2% after dance) after the first dance of a day. However, this pattern did not reach statistical significance. In this regard, the breeding male was more commonly responsible for maintaining proximity to F21 than he was to F11, and therefore proximity after dance was not substantially different than proximity before dance. We note, however, that F21 spent more time in proximity to the male after her first dance bout in 4 of the 6 days in which F11 did not dance (data based on days in which we observed the group for more than 8 hours). These data are consistent with the expectations of H2A and H2B and indicate that cao vit gibbon females used dancing behavior to establish and maintain a social bond with the breeding male.

Finally, our results are consistent with H3, namely that the dance display in cao vit gibbons represents a non-aggressive form of intrasexual competition among resident females. In this regard we found that the non-preferred female danced more often, was more likely to engage in multi-dancing bouts, and that both females were more likely to dance if the other female had danced earlier (co-dancing bout.) Also co-dancing bouts were more likely to contain multiple dances than were individual dancing bouts (10/22 vs 59/220). This suggests an effect of contagion or that in response to the sight of one female dancing the other female danced. Further analyses showed that the female who danced more often spent more time in proximity to the male than the remaining female. This was true when we analyzed data within a single day or across an entire month. These results suggest that females may use dance to compete for short-term social and sexual access to the breeding male within the context of a “dance more than the other female” principle. This non-aggressive form of competition may serve to promote or enhance group stability in these bi-female breeding groups. In this regard, we observed only two cases of direct female-female aggression in G1, and no cases in G1A, G2 and G4 during 3000 h of field observations.

Dance in gibbons was characterized by a varied set of body gestures organized into movement sequences that occurred during fertile and nonfertile periods. These movement sequences appear to be different from the highly stereotyped and predictable courtship displays reported in many birds^5^,^7^. In addition, courtship displays in birds are limited to the breeding season^8^. Our results suggest that copulation and reproduction are not the sole purpose of female gibbon dance. Female gibbons may use dance as a social tool to establish and maintain an affiliative and sexual bond with the group’s breeding male, and possibly as a form of nonaggressive intrasexual competition for access to the resident male.

Recently, Wang^20^ proposed the rhythmic adaptation hypothesis to explain the biological origin (primary selection pressure) and social evolution (secondary selection pressure) of dance, music, and speech. He argued that the repetition of rhythmic movements represents an unambiguous signal indicating the motivation and intent of the sender of a message resulting in the evolution of a rhythm-related reward and emotional system^20^. In the case of primates, this may be associated with both mirror neurons and motor neurons in the premotor cortex, which is located in the frontal lobe of the brain^21^. Mirror neurons and motor neurons are activated either when an individual performs a particular motor action or when an individual observers another performing that action^22^. Fogassi and Ferrari^21^ argue that “the capacity of the motor system to voluntarily control goal-directions and links between the motor system and the perception of others’ actions and gestures have played a primarily role in the emergence of complex sensorimotor and cognitive capacities related to communication”, in particular gestures involving the hands and mouth.

Despite decades of study of hundreds of non-human primate species, a rhythmic dance display has so far only been reported in highly encephalized taxa such as gibbons (this study)^12^, siamangs^13^,^14^, African great apes^9^,^10^,^11^ and white-faced capuchin monkeys^15^. This nonverbal form of communication may require complex cognitive abilities associated with fine motor control, and interpreting the intent and motivation of the signaler.

Although controlled studies of gibbon cognitive abilities are limited, gibbons share with other apes a relatively large brain and an expansion of the cerebellum, compared to monkeys^22^. “In particular, the lateral cerebellum is larger in gibbons than in monkeys, which, among other functions, is activated during planning of movement and in visuo-spatial problem solving”^23^. The lateral cerebellum controls functioning of the motor and premotor cortex, is associated with the prefrontal cortex (functions in planning and decision-making), and serves to integrate movement and cognitive abilities^22^. The expansion of this region appears to represent a shared derived trait in hominoids and is consistent with the contention that apes engage in more complex movements and gestural communication than other nonhuman primates^11^,^14^,^22^,^24^.

All gibbon species produce a series of highly distinct loud calls in the early morning. These male-female duets are reported to serve several functions including announcing, maintaining, and reinforcing a pair bond^25^,^26^. Because of the complex rhythmic structure, gibbon loud calls are referred to as “songs”^27^. In addition, adult females of all gibbon species exhibit a ritualized locomotor display including rapid brachiation, a form of bimanual arm swinging, and branch-shaking at the climax of their song^25^,^27^. Thus gibbons engage in several highly coordinated visual, vocal, and movement displays, including the dance display described here, that serve an important role in communication and social bonding.

Human dance is associated with highly complex and varied rhythmic movements that are culturally constructed and thus differ from the dances performed by non-human primates. However, similarities in neural architecture among hominoids suggest continuity in function associated with the processing of the meaning of sequenced movements, vocal information, and symbolic thought^22^. In the case of gibbons, a greater understanding of the function of dance in social communication may offer new insights into our understanding of origin of human dance and language. It may also provide a comparative framework for examining differences and similarities in rituals and displays among nonhuman primates, other mammals, and avians.

## Methods

### Study site and study animals

The cao vit gibbon is a critically endangered primate species with fewer than 20 family groups remaining in the wild. These groups are distributed along a karst forest patch on the border between China and Vietnam^28^. This karst limestone area is characterized by steep-sloped sharp-peaked mountains surrounded by two branches of the Quay Son River that flow into Vietnam^28^. The area contains monsoon tropical forest that was severely degraded by selective logging, fuelwood collection, charcoal making, and agriculture^29^. Detailed descriptions of the study site have been reported in other papers^28^,^29^.

From December 2007 to December 2013 we systematically monitored the social dynamics of four groups (G1, G1A, G2, and G4) of cao-vit gibbons that range wholly or partly in China^18^,^19^. The breeding male in Group G1 was aggressively replaced by a new male in July 2012^19^ and after the replacement we refer to this as a new or fourth study group (G1A). We intensively studied each group’s ecology and social behavior from July 2008 to December 2009 (G1, G2 and G4) and from July 2012 to December 2013 (G1A and G4). G1 was observed for 1175 h across 171 days, G2 for 201 h across 67 days, G4 for 1026 h across 171 days, and G1A for 789 h over 129 days. Except for an old female (Female F22 who was probably more than 30 years old) in G2, the remaining five females in our study groups each gave birth to 2–3 infants (total 13 infants) over the 6-year study period^19^ (Table S1). The two 6-month old infants in G1 died during the male replacement event of unknown causes. One small juvenile died in G1 and one in G4^18^,^19^ in the absence of male replacement. All other gibbons in each of the four study groups survived and either remained in their group or dispersed^19^.

### Data collection

Due to the steepness of the karst landscape, traditional or standard primate observational techniques (i.e. direct follows) were not possible at this site. Therefore, we observed gibbons from several established observation posts at distances of between 50 and 500 m (most observations were made at a distance of 100–300 m), using 8 × 30 binoculars (Steiner safari) or a spotting scope (Leica Apo-Televid77 20–60)^19^,^29^. Average tree height equals 10.5 m^29^ and the gibbons always travelled and rested close to the tops of these small trees. Through our Leica telescope we were often able to obtain direct and clear views of the gibbons. Although using this observation method we are likely to have missed subtle changes in behavior (e.g. certain facial expressions or gestures), by observing from a distance we did not disturb gibbon behavior or the frequency and context of gibbon displays. During all observation periods we attempted to observe group members until all individuals entered their sleeping trees, however, full-day observations were extremely difficult^19^.

Given these observation conditions, focal animal sampling was impractical and therefore we used a scan sampling method at 5-min intervals to record the activity and inter-individual distance of all visible group members^19^,^29^. Once we observed one adult approaching (an adult male or an adult female approached the other to within a distance of 0–3 m) another, we used an all occurrence data collecting method to record social behavior (e.g. dancing display, copulation, grooming, embrace, aggression). We recorded the time of approach, the identity of the approaching individual, departure time, and the identity of the departing individual. Specifically designed for this study, we recorded the last behavior that we observed immediately prior to dance. If we observed a dance, we noted the start time and duration whenever possible, the identity of the individuals involved, and the reaction of other adult group members. Behavioral context pre-dancing was recorded as approaching, resting in proximity (≤1 m including mutual grooming), resting apart (>1 m), feeding, and singing. The adult male’s reaction to a female dancing was recorded as departure, no obvious reaction, approach, engage in grooming only (give or receive grooming from the female), engage in copulation only, and engage in copulation plus grooming. We also video recorded two complete dance displays performed by the same adult female in G1.

### Data analysis

We analyzed the two video recordings to describe in detail the characteristics of gibbon dance including the sequence of all movements of the limbs, torso and head, the time interval between changes in body movements (29 frames per second were recorded by the camera), as well and vocal displays emitted during the dance. We used a Contingency Table Chi-Square test to analyze whether adult males displayed different reactions to dancing females in different behavioral contexts.

Although we observed females dancing in each of the four study groups, the vast majority (91.7%) of dance bouts were observed in G1A, after the replacement of the breeding male. We compared the dancing frequency of the two females in G1 and G1A (before and after male replacement, same females but different breeding males) to document the function of dancing in the development of male-female bonds. For other analyses, however, sample sizes for groups G1, G2 and G4 were too small to analyze quantitatively (less than 11). Therefore, all subsequent analyses were conducted only on dancing bouts performed in G1A. For each of the two females in this group we calculated the monthly proportion of time spent resting in proximity (≤1 m) to the breeding male, and then calculated the overall mean for each male-female dyad. Given that we observed G1A for only 6 hours in October 2012, this month was excluded from analysis. We then calculated the Hinde index using the proportion of approaches by a female to the resident male relative to the total number of approaches between that female and male minus the proportion of withdraws by that female and male relative to the total number of withdraws between them^30^,^31^. A Hinde Index of −1.0 means that the male is 100% responsible for maintaining proximity to the female. A Hinde Index of +1.0 means that the female is 100% responsible for maintaining proximity to the male.

We combined proportions of proximity and the Hinde index to analyze partner preferences^31^. We defined the preferred female as the female for whom the adult male was most responsible for maintaining proximity to and spent the greatest proportion of his time in close proximity with. The female for whom the adult male was least responsible for maintaining proximity to and spent the lowest proportion of his time in close proximity with was defined as the non-preferred female.

We used a Chi-Square test to analyze whether (1) the male was more likely to copulate with the preferred female in the absence of dance compared to the non-preferred female, and (2) a female was more likely to perform a multi-dancing bout if the other female had danced in proximity to the male within the previous 2 minutes. We used a Mann-Whitney U test to compare dance and copulation frequency between periods when females were cycling or pregnant. We estimated conception as seven months prior to the date of birth of a female’s infant^32^. We used a Spearman correlation to analyze the relationship between dancing frequency and male-female proximity in the same month and in the subsequent month. Periods during which females were lactating (1 month for F11 and two months for F21) were excluded from this analysis because both females ceased dancing during lactation. We used a Wilcoxon Signed Ranks test to analyze whether females spent more time in proximity to the male after the first dance of that day compared to prior to dancing on that day. We analyzed Hinde index to see if a given female or the group’s lone adult male was more responsible for maintaining proximity to each other after dancing compared to prior to dancing on that day. To avoid the effect that dance by the other female might have on the spatial relationship between a male-female dyad, we only used data in which we observed only one female to dance and we observed the group for at least eight hours that day. In order to analyze whether a female who danced more often spent more time in proximity to the male than did the other female, we created a variable in which F11’s dance frequency was subtracted from F21’s dance frequency (V1), and then we subtracted F11’s% time in proximity to the male from F21’s% of time in proximity to the male (V2), and used a Spearman correlation to analyze the relationship between the two values. We conducted this analysis using data only for days in which we observed the group for more than eight hours and females either were cycling or pregnant. Statistical significance was set at p < 0.05. This research was permitted by the Bangliang National Nature Reserve, and adhered to the legal requirements of China. No gibbons were manipulated during the whole study.

## Additional Information

**How to cite this article**: Fan, P.-F. *et al.* Rhythmic displays of female gibbons offer insight into the origin of dance. *Sci. Rep.* **6**, 34606; doi: 10.1038/srep34606 (2016).

## Supplementary Material

Supplementary Information

Supplementary Video 1

## Figures and Tables

**Table 1:** Adult male’s response to female dancing in different behavioral contexts.

Female dancing context		Male reaction						
		Departure	No reaction	Approaching	Grooming	Copulation	Grooming and copulation	Total
Approaching	N	20	20	3	24	10	6	83
	%	24.1	24.1	3.6	28.9	12.0	7.2	100
Resting in proximity	N	19	25	0	24	23	7	98
	%	19.4	25.5	0	24.5	23.5	7.1	100
Resting apart	N	2	12	3	5	2	1	25
	%	8.0	48.0	12.0	20.0	8.0	4	100
Feeding	N	5	10	0	0	2	0	17
	%	29.4	58.8	0	0	11.8	0	100
Singing	N	4	13	1	0	1	0	19
	%	21.1	68.4	5.3	0	5.3	0	100
Total	N	50	80	7	53	38	14	242
	%	20.7	33.1	2.9	21.9	15.7	5.8	100.1
