# Supplementary material for: Rhythmic displays of female gibbons offer insight into the origin of dance
Source: Sci Rep. 2016 Sep 30;6:34606. doi: 10.1038/srep34606 (PMC5043361; doi:10.1038/srep34606)
Supplement: Supplementary Information [file srep34606-s1.pdf]

Title: Rhythmic displays of female gibbons offer insight into the origin of dance

Authors: Peng-Fei Fan[a,b,1*], Chang-Yong Ma[a,b,1], Paul A. Garber[c], Wen Zhang[b], Han-Lan Fei[b], Wen Xiao[b*]

a. *School of Life Sciences, Sun Yat-sen University, Guangzhou 510275, P.R. China*

b. *Institute of Eastern-Himalaya Biodiversity Research, Dali University, Yunnan 671000, P. R. China.*

c. *Department of Anthropology Program in Ecology and Evolutionary Biology, University of Illinois, Urbana, Illinois 61801, United States of America*

1. These authors contributed equally to this study.

*Corresponding author**:** Peng-Fei Fan

School of Life Sciences, Sun Yat-sen University, Guangzhou 510275, P.R. China

Tel: 86-139-295-45210

E-mail: fanpf@mail.sysu.edu.cn.

*Corresponding author: Wen Xiao

Institute of Eastern-Himalaya Biodiversity Research, Dali University, Yunnan 671000, P. R. China.

Tel: 86-138-872-17775

E-mail: xiaowen.dali@gmail.com

Table S1. Birth dates of 12 infants produced by five adult female cao vit gibbons in four bi-female groups in Bangliang Nature Reserve, China. We distinguished G1 from G1A after the breeding male was replaced in this group. *These two infants were born after male replacement.

Group	Female	First	Second	Third*
G1	F11	2008.10.30 ± 6 days	2011.12.10 ± 12 days	2013.12.4 ± 5 days
	F21	2008.11.30 ± 1 days	2011.12.10 ± 12 days	2013.11.3 ± 7 days
G2	F21	2009.2.20	2011.1.24	2013.12.
G4	F14	2008.11.16	2012.1.31 ± 13 days	
	F24	2010.7.10 ± 5 days	2013.3.19	

Video legend: A short video of dancing display performed by a female (F11) in the main study group.
